# A Simulation-based Randomized Controlled Study of Factors Influencing Chest Compression Depth

**DOI:** 10.5811/westjem.2015.9.28167

**Published:** 2015-11-13

**Authors:** Kelsey P. Mayrand, Eric J. Fischer, Raymond P. Ten Eyck

**Affiliations:** Boonshoft School of Medicine, Wright State University, Fairborn, Ohio

## Abstract

**Introduction:**

Current resuscitation guidelines emphasize a systems approach with a strong emphasis on quality cardiopulmonary resuscitation (CPR). Despite the American Heart Association (AHA) emphasis on quality CPR for over 10 years, resuscitation teams do not consistently meet recommended CPR standards. The objective is to assess the impact on chest compression depth of factors including bed height, step stool utilization, position of the rescuer’s arms and shoulders relative to the point of chest compression, and rescuer characteristics including height, weight, and gender.

**Methods:**

Fifty-six eligible subjects, including physician assistant students and first-year emergency medicine residents, were enrolled and randomized to intervention (bed lowered and step stool readily available) and control (bed raised and step stool accessible, but concealed) groups. We instructed all subjects to complete all interventions on a high-fidelity mannequin per AHA guidelines. Secondary end points included subject arm angle, height, weight group, and gender.

**Results:**

Using an intention to treat analysis, the mean compression depths for the intervention and control groups were not significantly different. Subjects positioning their arms at a 90-degree angle relative to the sagittal plane of the mannequin’s chest achieved a mean compression depth significantly greater than those compressing at an angle less than 90 degrees. There was a significant correlation between using a step stool and achieving the correct shoulder position. Subject height, weight group, and gender were all independently associated with compression depth.

**Conclusion:**

Rescuer arm position relative to the patient’s chest and step stool utilization during CPR are modifiable factors facilitating improved chest compression depth.

## INTRODUCTION

Following the First National Conference on Cardiopulmonary Resuscitation in 1966, the Journal of the American Medical Association published the initial iteration of “Standards for Cardiopulmonary Resuscitation (CPR) and Emergency Cardiac Care (ECC)” in 1974.^1^ Multiple periodic updates of these standards shifted emphasis toward a systems approach credited with helping some programs achieve significantly higher-than-average resuscitation rates by developing a comprehensive structure addressing each of the links in the chain of survival.^2^ The 2005 update of the guidelines stressed the importance of high-quality CPR with defined standards for compression rate, depth, recoil, and maximal acceptable time for interruptions in compressions.^3^ Supported by additional research,^4^–^8^ the 2010 guidelines further emphasized high-quality CPR as the “cornerstone of a system of care that can optimize outcomes beyond return of spontaneous circulation.”^9^ Recent studies support the relationship of high-quality CPR to improved clinical outcomes.^10^,^11^ These changes require a refocusing of priorities during resuscitation to assure that high-quality CPR is being provided. Resuscitation team leaders can track the time-dependent CPR standards with relative ease, but monitoring compression depth and recoil is more subjective. The quality of compressions during training can be assessed with various indicator devices incorporated within training mannequins. However, real-time feedback devices, both stand alone and attachments to newer monitor/defibrillators,^12^–^15^ are not currently available in many clinical settings. The demands of advanced life support interventions (e.g. medication doses, energy levels, and algorithms) can distract the attention of resuscitation teams away from chest compression quality. This was evident when our group recently assessed the impact of a backboard on compression depth achieved with CPR performed on a mannequin positioned on an emergency department (ED) gurney (manuscript in preparation). Although the subjects all successfully completed an Advanced Cardiac Life Support (ACLS) course in the previous six months, we found that the majority of subjects in both control and treatment groups failed to routinely lower the bed, use a step stool, focus attention on compression quality, or achieve the 50 mm compression depth advocated in the 2010 guidelines. Consistent with our experience, other researchers have identified that a significant percent of healthcare providers fail to achieve the recommended compression depth.^16^,^17^

**Figure 1:**
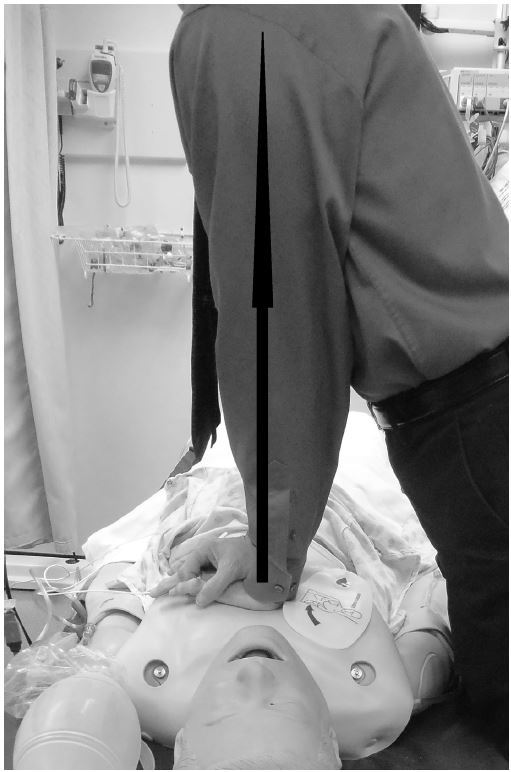
Proper position with the shoulders directly over the point of compression and the rescuer’s arms forming a 90° angle with the patient’s chest.

Variables that may influence compression depth include bed height, step stool utilization,^18^–^20^ the rescuer’s height,^18^ weight,^21^–^23^ and gender,^23^,^24^ and team focus on compression depth as an important aspect of resuscitation. One additional factor we observed was the position of the rescuer’s arms and shoulders relative to the point of compression over the chest. Ideally, the rescuer should position his/her shoulders directly over the point of compression so that the rescuer’s arms form a 90º angle to the patient’s chest (Figure 1). To assess these variables we conducted a high-fidelity mannequin study. Our research hypotheses included the following: 1) Using a stepstool and lowering the bed will significantly increase the mean compression depth; 2) Rescuers attaining a 90º angle with their arms and the mannequin’s chest (placing the shoulders directly over the point of compression) will achieve a greater mean compression depth; 3) Males will achieve a greater mean compression depth than females; and 4) Mean compression depth will increase with the rescuer’s height and weight.

## METHODS

Following approval by our university’s institutional review board, we recruited subjects from a cohort of 56 trainees including physician assistant (PA) students and first-year emergency medicine residents completing resuscitation practice as a required part of their respective curricula following completion of an ACLS course within the previous month. Each resuscitation scenario required at least one two-minute segment of CPR per American Heart Association (AHA) guidelines. Subjects were informed that automatically-recorded data from the SimMan Essential^TM^ (Laerdal, Norway) mannequin would be evaluated as part of a research project, but the nature of the data being assessed was not revealed to subjects.

We solicited the entire class of PA students and first-year emergency medicine residents to avoid selection bias. The condition for the subjects in the experimental group included setting the bed in the lowest position (64cm from the floor to the top of the mattress) with a step stool (23cm height) prominently placed next to the bed. For the control group, we placed a locking device in the bed, which elevated the lowest bed setting by 10cm (from 64cm to 74cm) and placed the stool under an IV cart at the foot of the bed so that it was available, but not prominently exposed. Ten cm elevation provided about 30% of the maximum bed height without making the modification readily apparent. We mounted a web camera on the wall at the foot of the bed in alignment with the center of the mannequin to record each subject’s shoulder position/arm angle relative to the compression point. The mannequin was placed on top of a CPR backboard on a standard 10cm foam mattress on an ED bed (Stryker Medical, Portage, MI).

Using a random number generator, we allocated groups of four subjects each to either the control or intervention condition. We used block randomization since the subjects completed the sessions in sets of four simulations. Prior to each session, we reviewed the Institutional Review Board-approved cover letter with all subjects, gave them a written copy and obtained their verbal consent for inclusion in the study. As each group entered the simulation lab, we recorded demographic data on an Excel spread sheet (Microsoft, Redmond, WA) including gender, height, and an estimation of each subject’s weight in one of three groups (<150lbs., 150–200lbs., or >200lbs.). We assessed the height using a measuring tape attached to the control room one-way mirror. Each height was a consensus measure by two of the investigators in the control room. The weight groups were arbitrarily selected to represent low, intermediate, and heavy weight ranges in a population of healthcare workers and each subject’s group was estimated by a consensus of the same two investigators. We instructed the subjects to complete all resuscitations in accordance with ACLS standards and to do everything they would do with an actual cardiac arrest patient. During the two-minute episodes of chest compressions, the mannequin software automatically recorded mean compression depth in 10-second segments.

A screen shot from each resuscitation video was captured during the beginning of the 2-minute compression period to assess each subject’s arm angle during compression. The screen shots were cropped providing a view from the top of each compressor’s shoulders to the mannequin’s chest without including facial or other identifying features. The screen shots were evaluated independently by two investigators who assessed the angle of the rescuer’s arm position relative to the mannequin’s chest as either 90° or less than 90°. The identity of the subject, the subject’s group, and their compression data was concealed. A third investigator served as the tiebreaker when the initial two assessments did not agree. Prior to the start of the study, the investigators were shown screen shots of compressors at a 90° angle and others at less than 90°.

### Statistics

We analyzed the results from the intervention and control groups and the arm angle of 90° and less than 90° groups with a 2-tail t-test for samples with equal variance using Excel™ (Microsoft, Redmond, WA) and reported these results as a mean with 95% confidence intervals. Using SAS version 9.4 (Cary, NC), the correlation between mean depth of compression and the subject’s height was assessed using a Pearson correlation coefficient (for two continuous variables), the correlation between compression depth and the subject’s weight group was assessed with a Spearman Rho correlation coefficient (for continuous and ordinal variables), and for gender with a point biserial correlation (for continuous and binary variables). We considered a p-value of <0.05 to be significant. We assessed the inter-rater reliability for assessing arm angle of 90° or less than 90° using Cohen’s kappa.

## RESULTS

**Figure 2:**
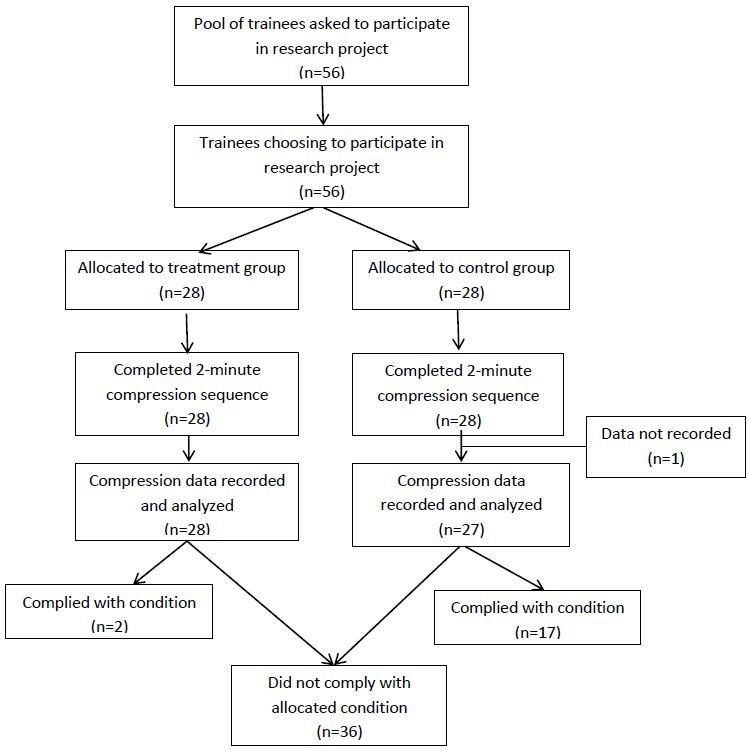
CONSORT flow diagram. *CONSORT,* Consolidated Standards of Reporting Trials

Fifty-six healthcare trainees verbally consented to participate in the study. Twenty-eight were randomly allocated to the intervention group and the other 28 to the control group. A complete data set was not recorded for one subject in the control group due to malfunction in the recording program and all reported results were derived with the data from the remaining 55 subjects (Figure 2).

**Figure 3:**
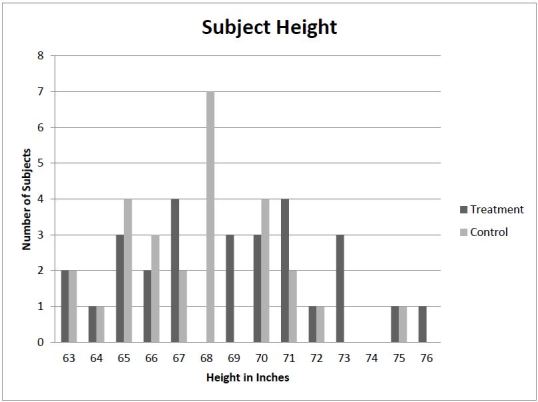
Distribution of subjects by height.

Thirty-five of the subjects were female and 20 were male. Twenty-six were in weight group 1, 21 in group 2, and 8 in group 3 (Table). Subject height ranged from 63 to 76 inches (Figure 3).

### Primary End Point

We compared the mean compression depth achieved in the intervention group and the control group using an intention to treat analysis. The intervention group achieved a mean compression depth of 39.3 (95% CI [35.4–43.2])mm compared to the control group 34.6 (95% CI [30.2–39.0])mm (p = 0.11).

Pre-positioning the step stool next to the bed in line with the mannequin’s chest was not associated with its use. Only two of 28 subjects in the intervention group used the step stool. Conversely, 10 of the 27 subjects in the control group found and used the step stool.

### Secondary End Points

**Figure 4:**
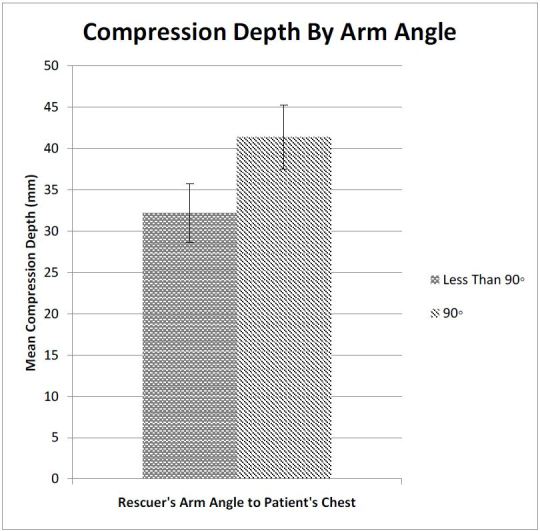
Mean compression depth, with 95% confidence intervals, of subjects who did and did not achieve a 90° arm angle.

The Cohen’s kappa for interrater agreement regarding arm angle/shoulder position was 0.87 indicating very good agreement between the two raters. The group of 29 subjects (18 from the intervention group and 11 from the control group) forming a 90° angle between their arms and the mannequin’s chest wall achieved a mean compression depth of 41.4 (95% CI [37.5–45.2])mm compared to 32.2 (95% CI [28.6–35.8])mm achieved by the 26 subjects in the group compressing at an angle less than 90° (p<0.003) (Figure 4). Post hoc analysis of the correlation between proper shoulder angle and the use of a step stool, using a Chi-squared test, revealed a significant correlation between using a step stool and achieving the correct shoulder position (p<0.02).

The correlations between compression depth and subject height, weight group, and gender were all statistically significant. The height and compression depth were strongly, positively correlated (Pearson correlation coefficient −r=0.560, p<0.0001). As height increases, compression depth also increases. The weight category and compression depth are strongly, positively correlated (Spearman Rho correlation coefficient −r=0.499, p=0.0001). As weight increases by category, compression depth also increases. Gender and compression depth are strongly correlated (point biserial correlation −r=0.499, p=0.0001). Mean compression depth for males is greater than that for females.

## DISCUSSION

Increased emphasis on quality chest compressions over the last 10 years has not translated to full compliance with current AHA recommendations. ACLS providers dedicate a great deal of mental energy to recalling algorithm sequences and drug doses, along with orchestrating the multiple time-sensitive, critical actions required from a frequently *ad hoc* team. Thus, suboptimal cardiac compression can potentially go undetected by a task-saturated team leader. When teams monitor chest compressions, they frequently focus on rate since it is the most readily detectable parameter to monitor. Various groups actively promote adjuncts like a metronome or a song rhythm (e.g. Stayin’ Alive) to support the recommended rate of at least 100 compressions per minute. Monitoring compression depth is more difficult. Devices providing immediate compression depth feedback are commercially available, but are currently not widely employed in clinical practice. In addition, most ACLS courses cannot provide the amount of chest compression practice needed for each learner to develop the conscious proprioception required to consistently recognize compression depths of >50mm. Each of the variables in our secondary end points correlated with improved compression depth. The height, weight, and gender of any particular rescuer cannot be modified, but the angle of the rescuer’s arms to the patient’s chest can easily be assessed during resuscitation and corrected, if needed, to achieve a 90° angle.

Even though the greater mean compression depth provided when the subject achieved a 90° angle was both statistically and clinically significant, the mean depth in this group was still nearly 9mm below the desired goal. However, of the 11 subjects attaining a mean compression depth of ≥50mm, 10 achieved a 90° angle. Increased emphasis on compression technique during training and testing sessions may be needed to reinforce the priorities advocated in current guidelines.

While we expected some of the intervention group subjects to ignore the step stool, we did not anticipate the higher utilization rate for the step stool in the control group. We concealed the step stool so it was not pre-positioned for use, but was available if sought in order to avoid drawing attention to the purpose of the study. In retrospect, some subjects performing chest compressions may have been motivated by the greater height of the bed in the control group to seek an adjunct to improve their position. Throughout the sessions, we rarely noticed the team leader or other team members addressing chest compression quality during a resuscitation scenario. The failure to consistently use adjuncts such as a step stool to improve chest compression mechanics suggests that ACLS providers do not prioritize chest compression depth relative to other cardiac arrest interventions.

## LIMITATIONS

There were a number of limitations to our study. First, in an attempt to employ a randomized design while masking the purpose of the study, we allocated subjects to conditions that we could not fully control. The bed height was well controlled, but the majority of subjects did not comply with the allocated condition for step stool use. The paradoxical increased use of a step stool in the control group potentially nullified some of the impact of the increased bed height. Consequently, it is difficult to conclude anything from the intention to treat analysis. Second, we did not calculate a sample size, but enrolled the entire class of PA students and residents during our orientation period. The resulting sample size was small including only 55 subjects. Third, our subjects were relatively inexperienced in running resuscitations even though they had all completed an ACLS course within a month of the study. However, less experienced providers are often the initial responders to cardiac arrests outside the ED and are responsible for running the first few minutes of an arrest, which is the most critical time if return of spontaneous circulation and a good functional outcome can be achieved. Fourth, although the subjects came from two different institutions and had completed ACLS training in two different courses, we cannot generalize our results to the wide spectrum of healthcare personnel completing advanced cardiac life support training.

## CONCLUSION

The majority of ACLS providers in our sample did not achieve the 50mm compression depth recommended by the American Heart Association. Subjects with an arm angle of 90° to the mannequin’s chest achieved significantly greater compression depth. The depth of compression was greater for males, as well as for taller and heavier subjects. We recommend ensuring a 90° arm angle during CPR to improve compression mechanics. Ensuring this arm angle provides a single simply-monitored factor that can be achieved by means of the rescuer’s physical characteristics, lowering the bed, using a step stool or some combination of these factors.

## Figures and Tables

**Table:** Demographics (gender and weight).

Gender	Weight group	Number of subjects	Intervention group	Control group
Male	1	1	1	0
	2	13	6	7
	3	6	4	2
Female	1	25	11	14
	2	8	4	4
	3	2	2	0
